# Quality control of opportunistic multi-energy CT bone mineral density quantification

**DOI:** 10.1007/s12194-026-01059-5

**Published:** 2026-05-07

**Authors:** Emily A. Thompson, Megan C. Jacobsen, Rachel M. Barbee, Jia Sun, Cayla A. Wood, Rick R. Layman

**Affiliations:** 1 Department of Imaging Physics, The University of Texas MD Anderson Cancer Center, 1515 Holcombe Blvd, 77030 Houston, TX USA; 2 Department of Biostatistics, The University of Texas MD Anderson Cancer Center, 1515 Holcombe Blvd, Houston, TX 77030 USA; 3 Department of Radiology, University of New Mexico, 2211 Lomas Blvd NE, Albuquerque, NM 87106 USA

## Abstract

**Background:**

Accurate assessment of bone mineral density (BMD) is crucial for evaluating bone loss in elderly and oncologic patients. Quantitative computed tomography (QCT) enables noninvasive quantification of patient BMD and can be performed opportunistically during routine CT examinations using either conventional or multi-energy CT (MECT). While both methods are used clinically, MECT can address limitations of conventional CT-based QCT.

**Purpose:**

This study addresses a critical gap in the literature by systematically evaluating commercially available calibration phantoms for use with MECT, assessing the impact of protocol variations, establishing a calcium-based material decomposition workflow for BMD quantification, validating phantom-derived metrics against patient data, and providing recommendations for integrating quality control (QC) into routine clinical workflows.

**Methods:**

Five CT QC phantoms containing bone-approximating materials, including calcium (Phantom A), dipotassium phosphate (Phantom B), hydroxyapatite (Phantom C), and calcium carbonate (Phantoms D and E), were evaluated by determining the dual-energy ratio (DER) of inserts. Phantoms were scanned on two identical dual-source CTs at 90/150Sn and 100/150Sn kVp with dose levels of 10 and 20 mGy. Images were reconstructed using filtered back-projection and iterative reconstruction. Phantom DERs were compared with retrospective vertebral measurements from a 10-patient cohort.

**Results:**

Phantom A provided the most suitable representation of patient data, exhibiting a DER of 1.55 [95%CI:1.54–1.57] at 100/150Sn kVp, compared with 1.44 [95%CI:1.37–1.50] in patients. kVp significantly influenced response, whereas radiation dose and reconstruction approach had minimal effect.

**Conclusions:**

Phantom selection is critical for MECT-based QCT, and calcium-based phantoms are well-suited for clinical QC and BMD quantification workflows.

## Introduction

Bone mineral density (BMD) is a measure of bone health and is used worldwide as a diagnostic metric for skeletal diseases, including low bone mass and osteoporosis, which are associated with increased fracture risk and mortality [1]. Low BMD affects millions of people worldwide and is a global public health concern [2, 3]. Dual-energy X-ray absorptiometry (DXA) is the imaging-based gold standard for measuring BMD [4]. DXA is widely adopted because of its low overall cost and radiation dose. However, the dose and cost advantages of DXA are largely negated when BMD measurements are obtained opportunistically from a clinically indicated standard-of-care computed tomography (CT) examination. In such cases, BMD values can be obtained from nearly any CT examination that includes the relevant anatomy (e.g., lumbar spine, pelvis) without additional radiation exposure.

Compared with DXA, CT offers several technical advantages, including volumetric (3D) bone imaging, which reduces overlap from adjacent anatomy and can improve sensitivity to changes in bone density [5, 6]. Quantitative computed tomography (QCT) development began in the 1970s [7, 8], and it is now an established clinical technique for noninvasive quantification of BMD [9, 10]. QCT analysis techniques can also be applied opportunistically to acquire BMD measurements from routine CT exams without additional patient radiation exposure or screening examinations [11]. Early QCT investigations relied on conventional single-energy CT (SECT) and external calibration phantoms to convert CT numbers to BMD values [12]. These studies demonstrated that energy-dependent attenuation, beam-hardening artifacts, metal implants, and partial volume averaging can adversely affect measurement accuracy [13, 14]. Quantitative SECT is also sensitive to patient size and variations in acquisition parameters, such as kVp [15, 16]. Furthermore, accurate BMD quantification using SECT is not feasible on contrast-enhanced CT examinations, as iodinated contrast material can artificially increase measured BMD values [17, 18].

More recent studies have investigated the use of multi-energy CT (MECT) for BMD estimation [19]. MECT acquisitions generate low- and high-energy datasets that can be combined to produce material-specific image reconstructions. Because the energy-dependent attenuation properties of different materials are known, MECT data can be decomposed into basis material images that separate calcium or iodine from soft tissue and fat. Basis-material decomposition has the potential to reduce the partial-volume effects inherent to SECT [20]. MECT’s ability to remove attenuation contributions from adipose tissue is particularly relevant for BMD assessment, since conventional QCT analyses that include marrow adipose tissue can introduce significant inaccuracies in BMD measurements [21].

Robust quality control and calibration are necessary for accurate QCT, regardless of acquisition technology. Historically, both synchronous and asynchronous calibration approaches have been investigated [22]. Several quality control and calibration phantoms are commercially available and incorporate inserts of known density, typically constructed from calcium carbonate (CaCO_3_), dipotassium phosphate (K_2_HPO_4_), calcium hydroxyapatite (CaHA), or other bone-surrogate materials suspended in epoxy resin [23]. The concentration of each material may be reported as either a calcium- or CaHA-equivalent concentration for use in calibration. However, most available phantoms were designed for use with SECT and include materials in the resin background matrix that result in erroneous material decomposition-based calcium or hydroxyapatite measurements on MECT. As a result, some commercially available BMD phantoms may not adequately characterize the energy-dependent attenuation behavior required for calibration of MECT-based BMD assessment. Currently, there is no universally accepted MECT-based QCT calibration phantom. The lack of comprehensive data regarding phantom validation is a fundamental barrier to the widespread clinical adoption of quality control (QC) in MECT-based QCT. This study addresses a critical gap in the literature by evaluating five commercially available calibration phantoms composed of various bone-surrogate materials for use with MECT, establishing a framework for using calcium as a basis material for material decomposition, validating phantom-derived results with patient data, and providing recommendations for integrating quality control into routine clinical workflows.

## Methods and Materials

### Phantom Selection

**Fig. 1:**
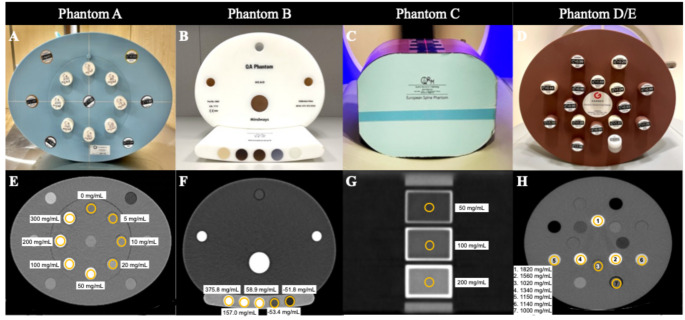
Photograph and CT image of each phantom. A/E) Phantom** A** Multi-Energy CT Phantom. B/F) Phantom** B** QCT Pro. C/G) Phantom** C** European Spine Phantom. D/H) Phantom** D** Electron Density/Dual Energy Phantom Model 467. Phantom E is not pictured as it is similar in appearance to Phantom D.

Five commercially available phantoms were evaluated for use with MECT and include the Multi-Energy CT Phantom (Mirion Sun Nuclear, Melbourne, FL) [Phantom A], QCT Pro (Mindways Software, Inc., Austin, TX) [Phantom B], European Spine Phantom (QRM, Möehrendorf, Germany) [Phantom C], Electron Density Phantom Model 465 (Gammex RMI, Melbourne, FL) [Phantom D], and Electron Density/Dual Energy Phantom Model 467 (Gammex RMI, Melbourne, FL) [Phantom E]. Descriptions of the phantoms are provided in Table 1. All phantoms include bone surrogate inserts (i.e., calcium (Ca), K_2_HPO_4_, CaHA, CaCO_3_) to quantify BMD at clinically relevant concentrations. Figure 1 shows each phantom and corresponding CT image with relevant inserts and concentrations.

**Table 1:** Phantom, material, and concentration details

Phantom	Material (or equivalent approximation) [24,25]	Nominal Concentration/Physical Density [23–25]
Phantom A: Multi-Energy CT Phantom	Calcium (Ca)	0, 5, 10, 20, 50, 100, 200, and 300 mg Ca/mL
Phantom B: QCT Pro Phantom	Dipotassium Phosphate (K_2_HPO_4_)	375.8, 157.0, 58.9, -53.4, -51.8 mg K_2_HPO_4_/mL
Phantom C: European Spine Phantom	Calcium Hydroxyapatite (CaHA)	Trabecular bone: 50, 100, 200 mg HA/mL Cortical bone: 400, 800 mg HA/mL
Phantom D: Electron Density/Dual Energy Phantom Model 467	Tissue Mimicking Materials Bone: Inner Bone: Bone Mineral (B200) Bone: CB2 (30% CaCO_3_) Bone: CB2 (50% CaCO_3_) Bone: Cortical (SB3) Water: Pure Water: Solid	1140 g/cm^3^ 1150 g/cm^3^ 1340 g/cm^3^ 1560 g/cm^3^ 1820 g/cm^3^ 1000 g/cm^3^ 1020 g/cm^3^
Phantom E: Electron Density Phantom Model 465	Tissue Mimicking Materials Bone: Cortical Bone: Inner Water: Pure Water: Solid	1840 kg/m^3^ 1145 kg/m^3^ 1000 kg/m^3^ 1015 kg/m^3^

### Scan Protocol and Image Analysis

All phantoms were scanned using a dual-source DECT system (SOMATOM Force, Siemens Healthineers, Forchheim, Germany). Conventional SECT and MECT scans were performed using the protocols shown in Table 2. The kVp, mAs, and reconstruction techniques were varied to evaluate the influence on CT number accuracy. Scans were repeated six times, three in each craniocaudal and caudocranial direction, to eliminate directional bias. Images were reconstructed at 1.5 mm slice thickness with a 1.0 mm interval using the Qr40 kernel with filtered back-projection (FBP) and iterative reconstruction (ADMIRE, Siemens Healthineers) at strength 3 out of a maximum of 5. These reconstruction settings simulate clinical conditions for opportunistic BMD measurement. Low- and high-energy images were subsequently reformatted to 5.0 mm slice thickness and 5.0 mm slice interval on a thin client server (syngo.via, VB30A_HF08; Siemens Healthineers). The mean CT number of each bone-equivalent phantom insert was measured using the methodology described by Thompson et al. [26]. Using custom-built semi-automated software (Matlab, R2023b), cylindrical volumes of interest (VOIs) with a height of 30 mm and a radius of 10 mm (volume: 9.425 cm^3^) were placed within each reference insert, with the cylinder axis aligned with the scanner’s z-axis. The mean and standard deviation of the mean CT number in the VOI were reported for SECT and low- and high-energy images across all acquisitions. Low- and high-energy image data were further processed as described in the next section.

**Table 2:** CT scan protocol for conventional SECT and MECT scans

kVp	Collimation	Rotation Time (s)	Pitch	mAs	CTDI_vol_ (mGy)
100	96 × 0.6 mm	0.28	0.6	250	10
				498	20
120	96 × 0.6 mm	0.28	0.6	150	10
				299	20
140	96 × 0.6 mm	0.28	0.6	102	10
				205	20
90/150Sn*	32 × 0.6 mm	0.28	0.6	172/108*	10
				344/215*	20
100/150Sn*	32 × 0.6 mm	0.28	0.6	148/74*	10
				296/148*	20

### Dual-Energy Ratio Computation

The dual-energy ratio (DER) is used in image-based material decomposition to classify a given material and generate material-specific images during post-processing [27]. The mean CT number for each VOI containing simulated bone or background (0 mg/mL) inserts was calculated. The DER is the slope derived from high- and low-energy images using linear least-squares regression [28].

### Patient Selection and Imaging

The Institutional Review Board approved a retrospective study of patient data. A review of the institutional EMR identified a random cohort of 10 patients (5 male, 5 female) who underwent both a dual-energy CT of the lumbar spine and DXA exam within 6 months. Patient ages ranged from 42 to 90 years (median age = 64), and each had been scanned using a clinical MECT protocol with 100/150Sn kVp, 32 × 0.6 collimation, 0.28 s rotation time, 0.6 pitch, and 360/180 quality reference mAs (tube A/tube B). Images were reconstructed with a slice thickness of 1.5 mm and an interval of 1.0 mm using a Qr40 kernel. High- and low-energy data were extracted from manually placed ROIs on vertebrae L1-L4, and the DER was calculated as described above using linear least-squares regression of low- and high-energy HU, plotted on a per-patient and per-vertebra basis.

### Material Decomposition and Calibration Curve

Phantom images were processed using a material decomposition profile customized to the DER of phantom A (syngo.via CT Dual Energy Virtual Unenhanced; version VB30A_HF08; Siemens Healthineers), with soft tissue and calcium as the basis materials, and the calcium DER set to 1.55. Adipose tissue was not incorporated in the material decomposition because the phantom inserts do not include a fatty component. Using the method of applying VOIs described above, calcium signal (HU) values were extracted from calcium-specific images and used to create a calibration curve of calcium concentration (independent variable) and calcium material decomposition signal (dependent variable). The mean and standard deviation were determined for each VOI.

### Statistical Analysis

For SECT images, the relationship between calcium concentration and CT signal (HU) was defined using linear regression. Differences in regression slope across acquisition conditions were evaluated using ordinary one-way analysis of variance (ANOVA) followed by Tukey’s post hoc test, using Phantom A as a representative example [29]. For DER calculations, linear regression was used to estimate the slope and y-intercept, and 95% confidence intervals (CI), R^2^ value, and root mean square errors (RMSE) were calculated. A one-way ANOVA followed by Tukey’s test was performed to evaluate differences among acquisition conditions in phantoms. Phantom attenuation response was compared with patient data by calculating the ratio of high- to low-energy HU at each concentration, followed by one-way ANOVA and Dunnett’s post hoc test to evaluate differences relative to vertebral measurements. Linear regression was used to model the relationship between calcium concentration and calcium-specific signal to generate a calcium calibration curve. All statistical analyses were conducted using a family-wise significance level of α = 0.05 (GraphPad Prism, v10.6.1).

## Results

### Scan Protocol and Image Analysis

Table 3 illustrates how specific SECT scan parameters influence calcium signals in Phantom A. One-way ANOVA with Tukey’s multiple comparisons test revealed that tube potential was the main factor causing variation, with data at 100, 120, and 140 kVp showing adjusted p-values < 0.0001 for all pairwise comparisons between kVp levels. Conversely, there were no significant differences between dose levels (10 vs. 20 mGy) or reconstruction approaches (FBP vs. ADMIRE) within the same kVp, as all these comparisons had 95% confidence intervals that spanned zero and adjusted p-values > 0.05. These findings suggest that calcium concentration measurements were strongly influenced by tube potential, while dose and iterative reconstruction had minimal impact under the conditions studied.

**Table 3:** Influence of kVp, CTDIvol, and reconstruction approach on the CT signal vs. calcium concentration relationship when evaluated with conventional SECT

kVp	Reconstruction	CTDIvol (mGy)	Slope [95% CI]	Y-intercept [95% CI]
100	ADMIRE	10	3.10 [3.09, 3.10]	3.77 [3.60, 3.94]
100	FBP	10	3.10 [3.10, 3.10]	3.84 [3.66, 4.02]
100	ADMIRE	20	3.11 [3.10, 3.11]	3.44 [3.27, 3.62]
100	FBP	20	3.11 [3.10, 3.11]	3.46 [3.28, 3.64]
120	ADMIRE	10	2.69 [2.69, 2.70]	2.60 [2.38, 2.81]
120	FBP	10	2.70 [2.69, 2.70]	2.61 [2.39, 2.83]
120	ADMIRE	20	2.69 [2.69, 2.70]	2.42 [2.26, 2.58]
120	FBP	20	2.69 [2.69, 2.70]	2.43 [2.26, 2.60]
140	ADMIRE	10	2.44 [2.43, 2.44]	1.20 [0.986, 1.41]
140	FBP	10	2.44 [2.44, 2.45]	1.15 [0.922, 1.37]
140	ADMIRE	20	2.43 [2.43, 2.43]	1.66 [1.48, 1.84]
140	FBP	20	2.43 [2.43, 2.44]	1.65 [1.46, 1.84]

### Dual-Energy Ratio Computation

**Fig. 2:**
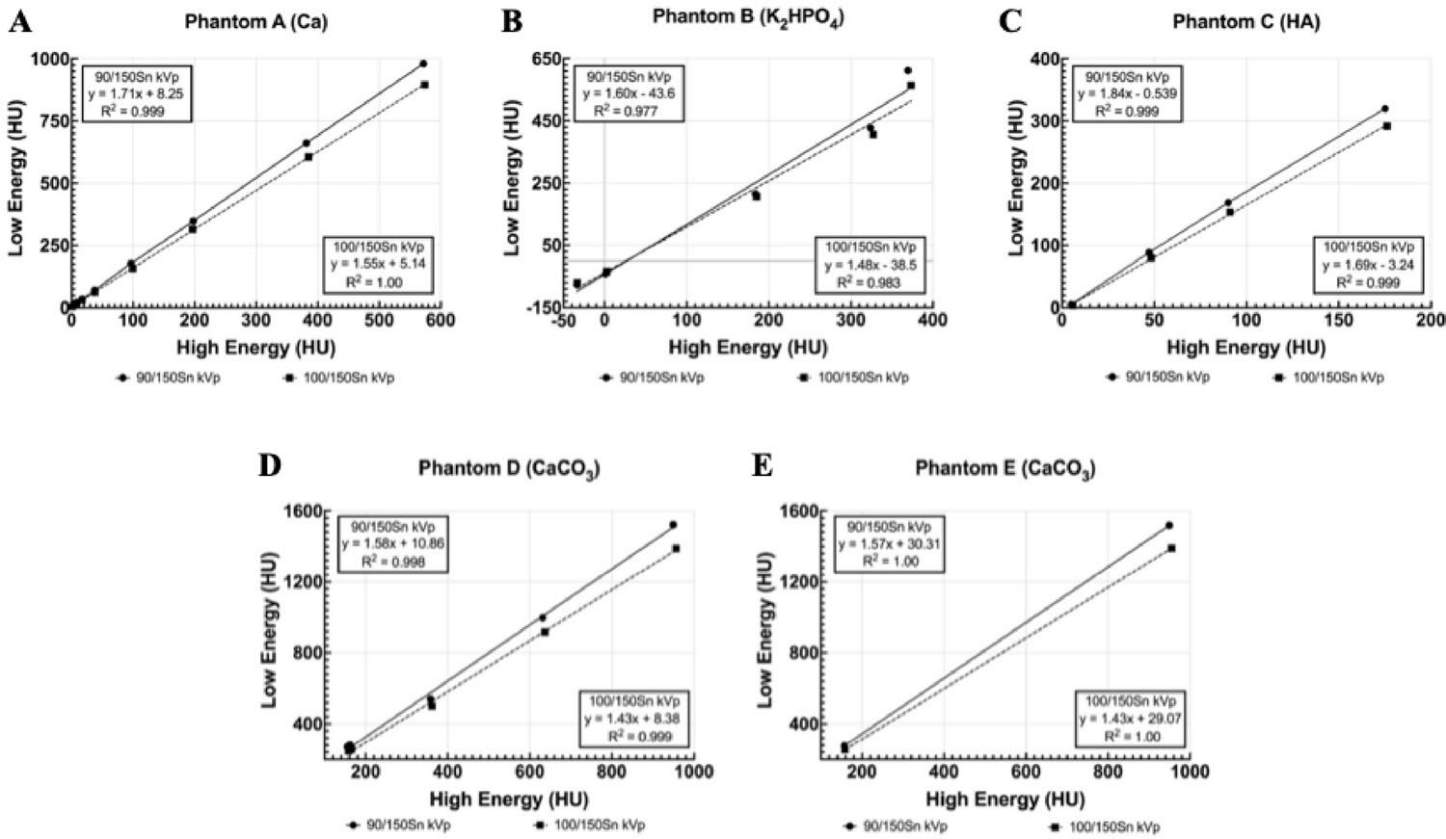
Dual-Energy Ratio (DER) for all phantoms acquired on a dual-source CT system. DER is equivalent to the slope of the plots shown. The 95% confidence interval is shown in brackets.** A** The DER for phantom A at 90/150Sn is 1.71 [1.69, 1.72], R^2^ = 0.999, RMSE = 3.11, and at 100/150Sn is 1.55 [1.54, 1.57], R^2^ = 1.00, RMSE = 2.43.** B** The DER for phantom B at 90/150Sn is 1.60 [1.15, 2.01], R^2^ = 0.977, RMSE = 52.25, and at 100/150Sn is 1.48 [1.11, 1.84], R^2^ = 0.983, RMSE = 41.68. **C** The DER for phantom C at 90/150Sn is 1.84 [1.71, 1.98], R^2^ = 0.999, RMSE = 4.13, and at 100/150Sn is 1.69 [1.56, 1.81], R^2^ = 0.999, RMSE = 3.74. **D**The DER for phantom D at 90/150Sn is 1.58 [1.46, 1.70], R^2^ = 0.998, RMSE = 25.32, and at 100/150Sn is 1.43 [1.35, 1.52], R^2^ = 0.999, RMSE = 18.45. **E** The DER for phantom E at 90/150Sn is 1.57 [y = 1.57x + 30.31], R^2^ = 1.0, RMSE = N/A, and at 100/150Sn is 1.43 [y = 1.43x + 29.07], R^2^ = 1.0, RMSE = N/A. For phantom E, the equation of the line is shown in lieu of the 95% confidence interval, as only two points are available for analysis. Error bars represent the standard deviation across six CT acquisitions and are smaller than the marker symbols. The 95% confidence interval is indicated in brackets.

As shown in Figure 2, the DER for the calcium inserts in phantom A is 1.71 [1.69, 1.72], R^2^ = 0.999, RMSE = 3.11 at 90/150Sn kVp and 1.55 [1.54, 1.56], R^2^ = 1.00, RMSE = 2.43 at 100/150Sn kVp. For phantom B, the DER at 90/150Sn is 1.60 [1.15, 2.01], R^2^ = 0.977, RMSE = 52.25, and at 100/150Sn is 1.48 [1.11, 1.84], R^2^ = 0.983, RMSE = 41.68. For phantom C, the DER at 90/150Sn is 1.84 [1.71, 1.98], R^2^ = 0.999, RMSE = 4.13, and at 100/150Sn is 1.69 [1.56, 1.81], R^2^ = 0.999, RMSE = 3.74. Inserts in phantom B showed bias in measured CT numbers at low concentrations, with the lowest-concentration insert measuring approximately − 65 HU at 100 kVp and − 30 HU at 150 Sn kVp. The DER for phantom D is 1.58 [1.46, 1.70], R^2^ = 0.998, RMSE = 25.32 at 90/150Sn and 1.43 [1.35, 1.52], R^2^ = 0.999, RMSE = 18.45 at 100/150Sn. The DER for phantom E is 1.57 [y = 1.57x + 30.31] at 90/150Sn and 1.43 [y = 1.43x + 29.07] at 100/150Sn. Note that, because phantom E contains only two bone-surrogate inserts, the DER yields a perfect linear fit between the two points. The equation of the line is shown instead of the 95% confidence interval, which cannot be calculated.

Table 4 shows the effect of selected protocol parameters on the DER for phantom A. As in conventional SECT (Table 3), ANOVA with Tukey’s multiple comparisons (α = 0.05) demonstrated a strong dependence on tube potential. All 90/150 kVp conditions had significantly higher mean DER values (1.70–1.71) than all 100/150 kVp conditions (1.55), with mean differences of 0.15–0.17 (95% CI: -0.19, -0.11, adjusted *p* < 0.0001). In contrast, no significant differences were observed within a given tube potential when varying the reconstruction approach or dose (10 vs. 20 mGy), as all adjusted p-values were ≥ 0.9770 and confidence intervals included zero. These results indicate that kVp is the primary driver of the observed differences, whereas reconstruction algorithm and dose have negligible effects within each kVp setting.

**Table 4:** Influence of kVp, CTDIvol, and reconstruction approach on the DER of phantom A

kVp	Reconstruction	CTDIvol (mGy)	Slope [95% CI]	Y-intercept [95% CI]
90/150Sn	ADMIRE	10	1.71 [1.69, 1.73]	8.25 [3.04, 13.45]
90/150Sn	FBP	10	1.71 [1.69, 1.73]	8.51 [2.93, 14.10]
90/150Sn	ADMIRE	20	1.71 [1.69, 1.72]	8.25 [4.75, 11.76]
90/150Sn	FBP	20	1.70 [1.69, 1.72]	8.29 [4.72, 11.87]
100/150Sn	ADMIRE	10	1.55 [1.53, 1.57]	4.47 [-0.93, 9.86]
100/150Sn	FBP	10	1.55 [1.53, 1.57]	4.49 [-1.03, 10.0]
100/150Sn	ADMIRE	20	1.55 [1.54, 1.57]	5.14 [2.40, 7.87]
100/150Sn	FBP	20	1.55 [1.54, 1.56]	5.15 [2.72, 7.58]

### Results from Patient Vertebra

**Fig. 3:**
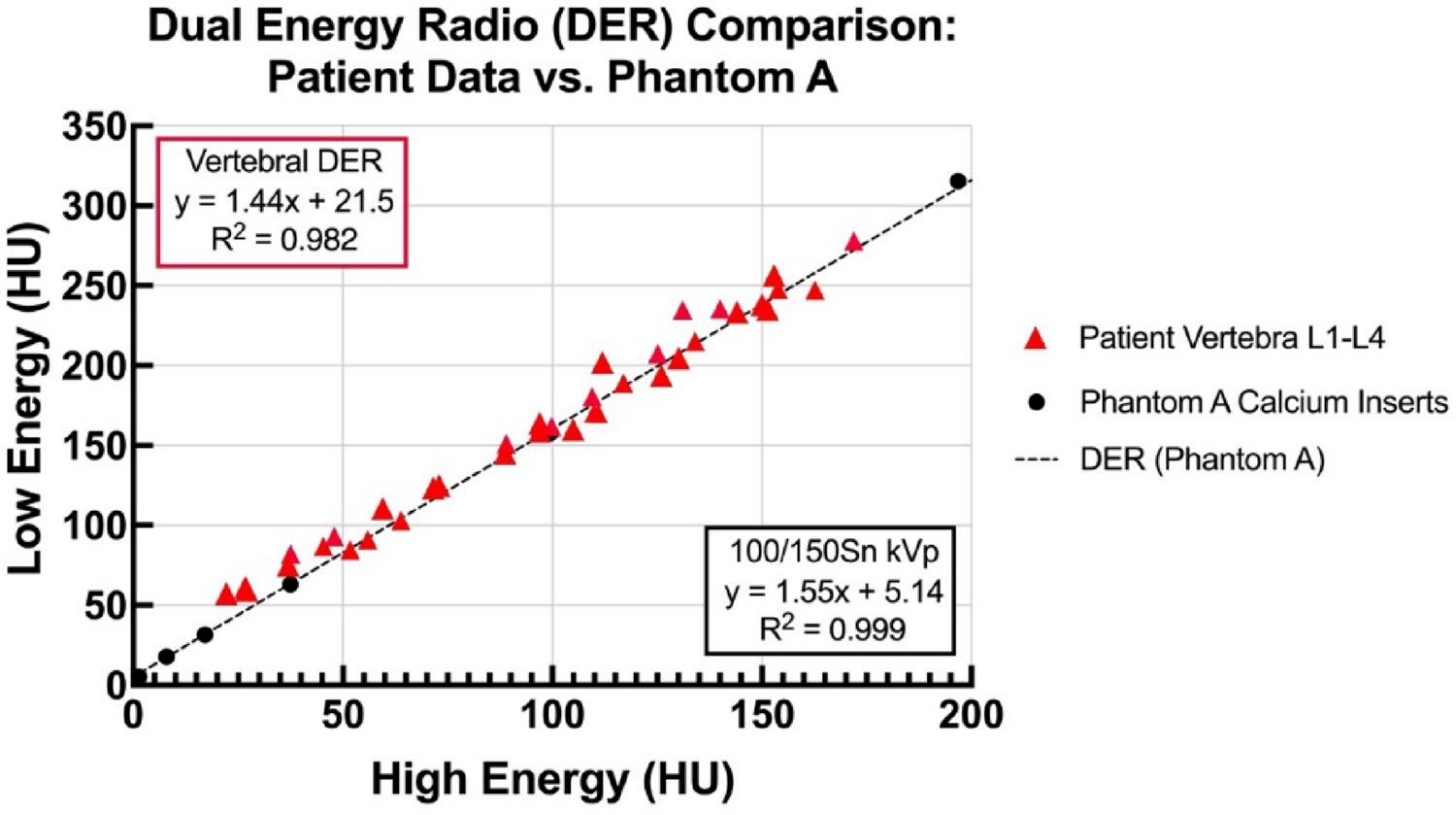
Dual-Energy Ratio (DER) for phantom A compared with the DER calculated from measurements in the L1-L4 vertebrae of 10 patients scanned with the same protocol. The DER calculated from vertebral data acquired at 100/150Sn is 1.44 [95% CI: 1.37, 1.50], with R^2^ = 0.982 and RMSE = 9.41.

The DER for patient vertebra was calculated and compared with the DER from each phantom scanned under similar conditions at 100/150Sn kVp. The DER calculated from vertebral data acquired at 100/150Sn is 1.44 [95% CI: 1.37, 1.50], with R^2^ = 0.982 and RMSE = 9.41 (Figure 3).

**Fig. 4:**
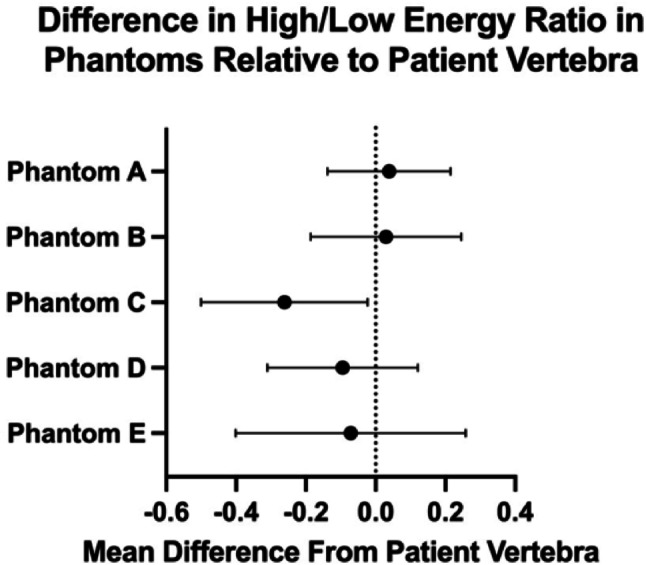
Plot of Dunnett-adjusted 95% confidence intervals for mean differences between the high/low energy (HU) ratio in patient data and each phantom at 100/150Sn kVp. The mean differences, with 95% confidence intervals, are shown relative to patient data (x = 0), with intervals crossing zero indicating no statistically significant difference after family-wise error rate correction (α = 0.05). The mean differences in DER value for each phantom were as follows, with brackets indicating the 95% confidence interval. Phantom A: 0.04 [-0.14, 0.21]; Phantom B: 0.03 [-0.19, 0.25]; Phantom C: -0.26 [-0.50, -0.02]; Phantom D: -0.09 [-0.31, 0.12]; and Phantom E: -0.07 [-0.40, 0.26].

A one-way ANOVA with Dunnett’s post hoc test was used to compare the phantom attenuation response with that of the patient vertebral reference data, using the ratio of high- to low-energy HU at each concentration. Mean differences ranged from − 0.26 to 0.04, with all 95% confidence intervals spanning zero. Phantom C demonstrated a statistically significant difference from patient data (mean difference = -0.26, 95% CI [-0.50, -0.02], adjusted *p* = 0.0253). Phantoms A, B, D, and E showed closer agreement with patient data (mean differences of 0.04, 0.03, -0.09, and − 0.07, respectively; adjusted p-values ≥ 0.7496) (Figure 4).

### Material Decomposition and Calibration Curve

**Fig. 5:**
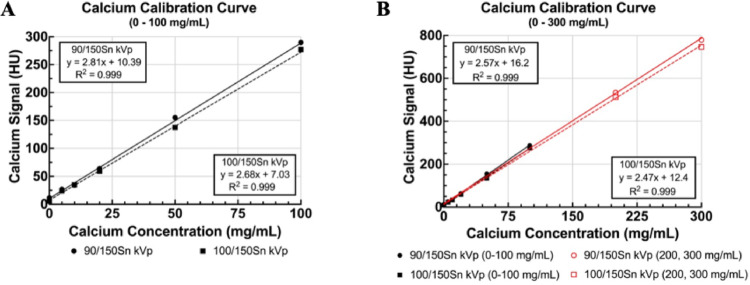
**A** The calcium calibration curve for Phantom A exhibited a slope of 2.81 [2.80, 2.82] and y-intercept of 10.39 [9.77, 11.00] at 90/150Sn kVp and a slope of 2.68 [2.67, 2.70] and y-intercept of 7.03 [6.50, 7.56] at 100/150Sn kVp.** B** Influence of high concentrations of calcium (200 and 300 mg/mL) that exceed the clinically relevant range on the calibration curve. Including the higher calcium concentrations shifts the calibration curve to a slope of 2.57 [2.56, 2.58] and y-intercept of 16.2 [15.0, 17.4] at 90/150Sn kVp and a slope of 2.47 [2.47, 2.48] and y-intercept of 12.4 [11.3, 13.4] at 100/150Sn kVp.

Figure 5 describes the calibration curve generated from the relationship between calcium concentration (mg/mL) and calcium signal (HU) values in Phantom A. Figure 5A shows this curve for a calcium concentration range of 0-100 mg/mL. In comparison, Figure 5B shows the influence of high calcium concentration (up to 300 mg/mL). High calcium concentrations that produce CT signal values near the maximum quantifiable threshold introduce a bias in calibration curves, as evidenced by the larger y-intercept values in Figure 5B.

## Discussion

This study evaluated five commercially available calibration phantoms composed of different bone-surrogate materials for use with MECT. The study was designed to assess phantoms based on their attenuation response at clinical MECT energies, using patient data to validate phantom results. Several studies have highlighted the importance of quality control in CT-based BMD quantification, and several phantoms and software packages are commercially available for use with both synchronous and asynchronous calibration in conventional CT [35]. However, to date, little has been published on the appropriateness or limitations of QC phantoms for MECT BMD applications. This study shows that conventional BMD phantoms can introduce bias and inaccuracies in BMD calibration. Using a phantom specifically designed for spectral CT is necessary for accurate calibration, as MECT-specific phantoms are constructed from materials that mimic the energy-dependent attenuation characteristics of human tissues across the energy spectrum used in MECT imaging.

MECT offers several advantages over SECT for BMD imaging, including reduced beam hardening, reduced metal artifacts, and material-specific quantification [30]. MECT enables the execution of material decomposition algorithms and the creation of calcium-specific images [31]. Calcium-specific images may increase the accuracy of BMD estimates by removing the influence of adipose tissue in the bone marrow, which can artificially lower CT numbers and, therefore, BMD [32]. This effect can be minimized with MECT by correctly identifying and quantifying a specific material within a given voxel [14]. The impact of adipose tissue on BMD estimates is particularly relevant to spine-based measurements [33]. MECT can also enable differentiation between cortical and trabecular bone, which is important as trabecular bone is more metabolically active and thus susceptible to early changes in density [34]. Another advantage of using spectral CT over conventional CT for BMD assessment is that three-material decomposition can be applied to remove contributions from iodinated contrast and adipose tissue, thereby enabling BMD quantification in patients undergoing contrast-enhanced CT studies.

Based on the results of this study, the MECT-developed calcium phantom (phantom A) was identified as the most appropriate surrogate for patient-derived DER representation because of its numerical agreement with the attenuation response measured via DER, high measurement precision, and a clinically relevant design spanning the range of calcium concentrations expected in patients. At 100/150Sn, phantom A exhibited a DER of 1.55 (95% CI [1.54–1.57]), with the narrowest confidence interval among all phantoms. The mean difference in the high/low energy ratio between phantom A and patient data was 0.04 (95% CI: -0.14, 0.21) and was not statistically significant (adjusted p-value = 0.9830). Phantom A also demonstrated the highest degree of linearity (R² = 1.000) and the lowest RMSE (2.43), indicating highly linear and reproducible behavior. Importantly, its multiple inserts spanned the range of calcium concentrations observed in the patient cohort, enabling representative calibration. Using the dual-energy response of calcium, a custom material decomposition profile was used to generate a calibration curve from which calcium concentration can be determined from the signal (HU) in calcium-specific images. This capability will be helpful for future applications in which calcium concentration must be directly determined from MECT images.

Phantom B demonstrated a DER of 1.48 (95% CI [1.11, 1.84]) but had the largest RMSE (41.68) among the phantoms, indicating substantial variability in the data. In addition, the negative y-intercept indicates a bias in the data, suggesting that the phantom background material is not a suitable surrogate for soft tissue at the energies used in diagnostic MECT. Phantom C exhibited the most significant deviation from the patient-derived DER (DER = 1.69 (95% CI: 1.56, 1.81)), with a statistically significant mean difference in the high/low energy ratio of -0.26 (adjusted p-value = 0.0253), and was therefore deemed unsuitable. Phantoms D and E yielded the same DER values at 100/150Sn kVp, but differed in their concentration-specific high/low energy ratios. The mean differences relative to patient data were − 0.09 (95% CI: -0.31, 0.12) for phantom D and − 0.09 (95% CI: -0.40, 0.26) for phantom E. Phantom D also exhibited a relatively large RMSE (18.45) in DER, while phantom E was excluded from comparative analysis entirely because its DER was derived from only two data points, precluding reliable estimation of uncertainty. Furthermore, neither phantom D nor E included more than two inserts with HU values within the range observed in the patient cohort, rendering both phantoms unacceptable for clinical application.

Bone-mimicking phantom inserts should be constructed with calcium or other compounds that accurately represent the attenuation characteristics of bony anatomy. Traditional substitutes, such as dipotassium phosphate (K_2_HPO_4_), commonly used as a bone substitute in conventional CT phantoms, may not be appropriate for MECT, as they do not accurately reflect the attenuation response observed in patients. This is evidenced by the relatively large RMSE for measurements taken in Phantom B (K_2_HPO_4_), indicating a nonlinear relationship between attenuation at high and low energies. The concentrations used in the phantom design should cover the clinically expected range of 0-150 mg Ca/ml [21, 36]. Phantom A included eight calcium rod inserts spanning the clinical range with a maximum calcium concentration of 300 mg/ml. Concentrations beyond this range will result in CT signal saturation and beam hardening artifacts at low kVp, introducing bias into calibration curves and subsequent BMD estimates.

Phantom background material is another important consideration when selecting phantoms for use with MECT. Phantoms designed for spectral CT applications are composed of background materials that exhibit similar attenuation across the energy spectrum used in MECT imaging. Background materials in the phantom inserts (0 mg/mL calcium) should not contain calcium or potassium, as this will bias the measured calcium concentration. Among the phantoms included in this study, phantom A was the only phantom specifically designed for MECT and most closely matched the attenuation response observed in patients. This highlights the importance of matching phantom background materials to tissue properties for both calibration accuracy and the development of reliable quality control protocols.

Results showed that the tube voltage strongly influences material quantification in SECT and MECT. Other protocol parameters, such as radiation dose and image reconstruction approach, are unlikely to affect BMD quantification substantially, provided image quality is sufficient. Consequently, opportunistic BMD measurements should be performed only when phantom calibration data are available at the same tube voltage(s) used in the patient scan. A primary goal of image acquisition in QCT analysis is to minimize the extent to which non-anatomic factors affect calculated results. This is accomplished by establishing a consistent protocol that standardizes as many acquisition parameters as possible. While BMD calibration data would ideally be available at multiple dose levels for validation, maintaining additional calibrations can be burdensome for clinical staff and is of limited relevance when tube current modulation is employed in clinical CT examinations.

Phantom A is comprised of two components – a body phantom with a head phantom insert. Throughout this study, the body phantom was used. At least one study reports that the smaller head phantom insert provides more stable and accurate attenuation estimates than measurements in the body phantom [37]. However, that study used a prototype phantom with higher background attenuation than the commercially available model, so beam hardening affected CT numbers in the inserts more dramatically. Secondly, the prior study used a scanner with 80/140 kVp, and the low kVp beam may not be optimal for larger phantoms and patients. Consideration of clinical application is also important, as the body phantom provides scan conditions that more accurately represent those of abdominal and pelvic scans, where opportunistic BMD evaluation will be integrated into our clinical workflow.

There are several limitations to this study. First, the results are specific to dual-source systems from one manufacturer. There are various approaches to MECT, from system design to acquisition, and these differences could result in some variation [38, 39]. Additionally, spectral separation can affect the accuracy of material quantification, which varies across MECT technologies [38, 39]. The patient data sample size (*N* = 10) was small. However, the measured calcium concentrations are comparable to those reported in the literature and enabled correlation to phantom data. Many studies report calcium concentrations obtained from vendor or third-party software, but hydroxyapatite and calcium measurements can vary across vendors, necessitating cross-calibration of MECT systems [39, 40]. Independent validation using dedicated phantoms remains underutilized but is critical for ensuring reliable BMD quantification, particularly when proprietary software is unavailable or when comparing results across platforms.

## Conclusions

Accurate BMD assessment with MECT requires a phantom that replicates the energy-dependent attenuation characteristics of human bone. Among the phantoms evaluated, only Phantom A demonstrated consistent agreement with patient-derived attenuation data, highlighting its suitability for MECT calibration and quality control. Results show that tube voltage has the greatest impact on calcium quantification, whereas reconstruction method and radiation dose have minimal influence when image quality is sufficient. These findings emphasize the need for protocol-matched calibration and support the development of an MECT-specific quality control program. Establishing standardized phantom-based validation is essential for ensuring reproducible, vendor-independent BMD measurements in opportunistic QCT workflows.

## Data Availability

Data that support the findings of this study are available via reasonable request to the corresponding author.
